# Online HIV prophylaxis delivery: Protocol for the ePrEP Kenya pilot study

**DOI:** 10.3389/fpubh.2023.1054559

**Published:** 2023-02-24

**Authors:** Catherine Kiptinness, Paulami Naik, Nicholas Thuo, Rachel C. Malen, Julia C. Dettinger, Jillian Pintye, Maeve Rafferty, Edwin Jomo, Nicky Nyamasyo, Tony Wood, Paul Isabelli, Sarah Morris, David Hattery, Andy Stergachis, Daniel Were, Monisha Sharma, Kenneth Ngure, Melissa Latigo Mugambi, Katrina F. Ortblad

**Affiliations:** ^1^Partners in Health Research and Development, Center for Clinical Research, Kenya Medical Research Institute, Nairobi, Kenya; ^2^Department of Global Health, University of Washington, Seattle, WA, United States; ^3^Public Health Science Division, Fred Hutchinson Cancer Center, Seattle, WA, United States; ^4^Department of Biobehavioral Nursing and Health Informatics, Seattle, WA, United States; ^5^MYDAWA, Nairobi, Kenya; ^6^Audere, Seattle, WA, United States; ^7^Department of Pharmacy, University of Washington, Seattle, WA, United States; ^8^Jhpiego, Nairobi, Kenya; ^9^School of Public Health, Jomo Kenyatta University of Agriculture and Technology, Nairobi, Kenya

**Keywords:** PrEP, PEP, HIV prevention, telehealth, differentiated service delivery, implementation science, Kenya

## Abstract

**Background:**

Online pharmacies in Kenya provide sexual and reproductive health products (e.g., HIV self-testing, contraception) and could be leveraged to increase the reach of HIV pre-exposure and post-exposure prophylaxis (PrEP/PEP) to populations who do not frequently attend health facilities. To date, evidence is limited for operationalizing online PrEP/PEP delivery and the type of populations reached with this differential service delivery model.

**Methods:**

The ePrEP Kenya Pilot will deliver daily oral PrEP and PEP *via* MYDAWA, a private online pharmacy retailer, to clients in Nairobi for 18 months. Potential clients will obtain information about PrEP/PEP on MYDAWA's sexual wellness page and self-screen for HIV risk. Individuals ≥18 years, identified as at HIV risk, and willing to pay for a blood-based HIV self-test and PrEP/PEP delivery will be eligible for enrollment. To continue with online PrEP/PEP initiation, eligible clients will purchase a blood-based HIV self-test for 250 KES (~USD 2) [delivered to their setting of choice for 99 KES (~USD 1)], upload an image of their self-test result, and attend a telemedicine visit with a MYDAWA provider. During the telemedicine visit, providers will screen clients for PrEP/PEP eligibility, including clinical concerns (e.g., kidney disease), discuss self-test results, and complete counseling on PrEP/PEP use and safety. Providers will refer clients who self-test HIV positive or report any existing medical conditions to the appropriate services at healthcare facilities that meet their preferences. Eligible clients will be prescribed PrEP (30-day PrEP supply at initiation; 90-day PrEP supply at follow-up visits) or PEP (28-day supply) for free and have it delivered for 99 KES (~USD 1). We will measure PrEP and PEP initiation among eligible clients, PEP-to-PrEP transition, PrEP continuation, and implementation outcomes (e.g., feasibility, acceptability, and costs).

**Discussion:**

Establishing pathways to increase PrEP and PEP access is crucial to help curb new HIV infections in settings with high HIV prevalence. The findings from this study will provide evidence on the implementation of online pharmacy PrEP and PEP service delivery that can help inform guidelines in Kenya and similar settings.

## Introduction

While online pharmacies were initially available only in high-income countries, they have been increasing in many low- and middle-income countries (1). The scope of online pharmacies may include direct-to-client distribution of health products through online and mobile channels, digital health information, and remote consultations with health providers. In Kenya and other sub-Saharan African countries, HIV pre-exposure and post-exposure prophylaxis (i.e., PrEP and PEP, respectively) services are mainly delivered through HIV comprehensive care clinics at public sector health facilities. Often PrEP initiation and continuation at these facilities is poor, which can be attributed to client-level barriers, such as a lack of privacy, HIV-related stigma, and time spent traveling to and waiting at the clinics (2, 3), as well as provider-level barriers, such as competing treatment priorities, overcrowding, and a lack of PrEP and PEP knowledge (4, 5). Despite the availability of PrEP, new and diverse HIV prophylaxis delivery models are needed to overcome these barriers and enable individuals to select a delivery model that fits their preferences and facilitates continuation during their periods of HIV risk.

Online HIV prophylaxis delivery, defined as the ability to initiate PrEP or PEP using telemedicine visits with remote clinicians, at-home HIV testing, and PrEP/PEP medication delivery, has not been tested in Africa. However, during the COVID-19 pandemic, some core components of this model, such as telemedicine visits (6–10), at-home HIV testing (6, 9–12), and at-home medication delivery (9, 10), were implemented successfully in low and middle-income countries to maintain access to HIV prevention and treatment services when travel restrictions were in place. While these models helped maintain service delivery among clients already engaged in care at public facilities during the pandemic, they also have the potential to reach new clients at HIV risk not currently receiving or interested in HIV prevention services at public facilities. New technologies, like HIV self-testing (HIVST) and growing access to mobile devices and networks among individuals across income brackets in Africa (13, 14), further support the feasibility of an online HIV prophylaxis delivery model in the region.

Kenya is well-positioned to lead efforts to develop, test and implement an online model for HIV prophylaxis service delivery, which could potentially serve as a model for other countries. As of 2022, an estimated 300,000 people in Kenya initiated PrEP services, one of the largest initiation rates in the east and southern African region (15). Kenya also continues to cater to the treatment needs of over 1.4 million people living with HIV (16, 17). Finally, Kenya boasts strong tech development talent and a large middle class population (18, 19) that provides further potential support for developing such an innovative delivery model. This pilot study aims to design and test the first online PrEP/PEP delivery model in Africa. We hypothesize that an online PrEP and PEP delivery model will be feasible, acceptable, and safe and can be delivered at a reasonable cost in Kenya.

## Materials and methods

### Study setting

Nairobi, the capital of Kenya, is a dense urban city with a population of 4.3 million (20) and a population-level HIV prevalence of ~5% (21). For the pilot, we will collaborate with MYDAWA, Kenya's first licensed online pharmacy (https://mydawa.com). MYDAWA has developed technical and operational capabilities to tackle and overcome traditional supply chain challenges (e.g., inconsistent pricing of health care products, stock-outs, substandard products) and provide affordable access to a wide range of quality prescription and over-the-counter medicines and products delivered directly to the consumer quickly, confidentially, and conveniently. Additionally, MYDAWA's platform often attracts clients seeking sexual and reproductive health products, such as emergency contraceptive products and HIVST kits, who might also be interested in the online delivery of HIV prevention services.

### Design of the online HIV prophylaxis delivery care pathway

To design a care pathway for online PrEP/PEP service delivery, we adapted an existing one for the delivery of PrEP and PEP services at brick-and-mortar pharmacies in Kenya (22), then refined this with input from key stakeholders and findings from a discrete choice experiment (DCE). Specifically, at meetings with Kenyan PrEP implementation experts, researchers, and Ministry of Health officials, we presented the adapted care pathway, elicited stakeholder feedback, and refined the model as needed. We ultimately landed on a care pathway that utilizes a prescribing checklist—similar to that used in the brick-and-mortar pharmacy PrEP/PEP service delivery model (22)—to identify clients eligible for online PrEP/PEP services (i.e., at HIV risk with no medical conditions that might contraindicate PrEP/PEP safety), and refer clients that do not meet the checklist criteria to clinic-based PrEP services. We also conducted a DCE with ~800 potential online PrEP/PEP clients to elicit their preferences for different core components of the intervention (described below) (23). The analysis of the DCE findings is ongoing; once completed, we plan to meet again with key stakeholders to share our findings and further refine the care pathway for online PrEP/PEP service delivery.

### Pilot design and population

We will conduct a prospective, single-arm pilot study to test this novel online PrEP and PEP delivery model. MYDAWA clients will be eligible to participate in the study if they are ≥18 years old, self-identify as at risk for HIV, and meet the criteria for online PrEP/PEP delivery on the prescribing checklist (e.g., self-tested HIV-negative, no contraindications to PrEP). Clients who self-report having a history of liver disease, kidney disease, diabetes, or hypertension will not be medically eligible to receive PrEP through the study. In addition, clients reporting symptoms that might reflect acute HIV infection, including sore throat, headache, or fever after having sex without a condom in the last 30 days, will not be eligible to receive PrEP through this study. Pregnant or breastfeeding women will remain eligible for online PrEP and PEP services.

As MYDAWA's business is conducted solely through their English-language website and mobile phone application, clients need to have access to a smartphone or computer during the pilot duration and be able to read and understand English. Although MYDAWA currently offers delivery across all of Kenya, clients must provide a delivery address within Nairobi County for efficient delivery to be eligible for this study. Additionally, eligible MYDAWA clients must be willing to pay for blood-based HIVST and PrEP/PEP delivery. We have not limited the number of clients who can enroll in this research and access online PrEP and PEP services, as this is an implementation study, and these are two of our primary outcomes.

### Pilot procedures and data collection

**Figure 1:**
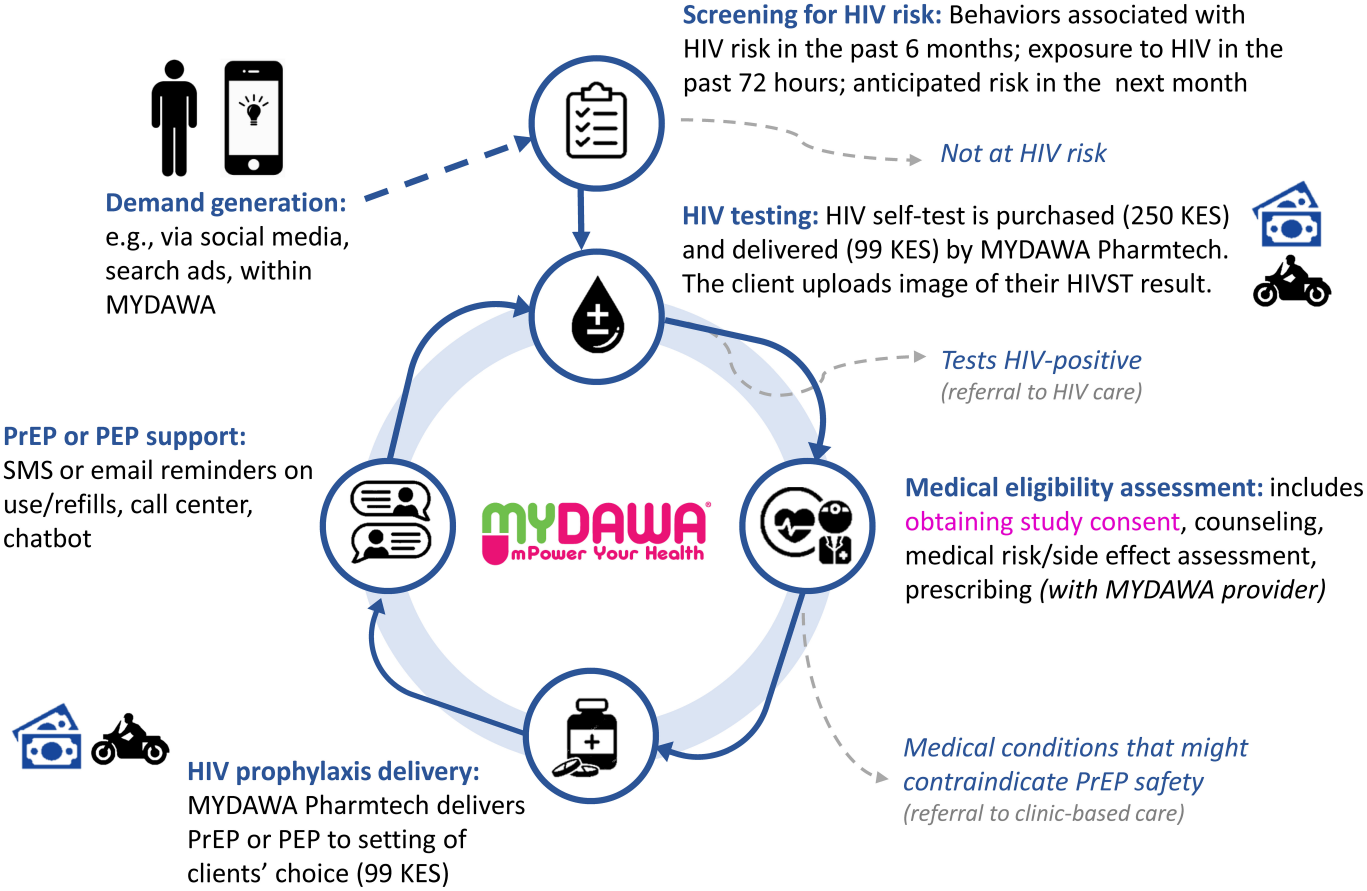
The proposed care pathway for online pharmacy PrEP or PEP service delivery *via* MYDAWA.

The care pathway for online HIV prophylaxis delivery consists of the following core components: (1) demand generation, (2) screening for HIV risk, (3) HIV testing, (4) medical eligibility assessment (*via* a telemedicine visit), (5) HIV prophylaxis delivery, and (6) PrEP or PEP support (see Figure 1). We describe each of these phases and associated procedures below.

#### Demand generation

We will advertise the online PrEP and PEP delivery model to potentially new and existing MYDAWA clients through multiple marketing channels, including social media campaigns (e.g., on Facebook and Instagram), search engine optimization for online searches of sexual and reproductive health products, and cross-promotion (i.e., targeting customers of other products and services sold by MYDAWA).

#### Screening for HIV risk

All MYDAWA clients interested in PrEP or PEP delivery must first complete an online self-screening of HIV risk or recent HIV exposure. Clients can access this online screening by following links on MYDAWA's sexual health and wellness page. We based this self-screening on Kenya's PrEP Rapid Assessment Screening Tool (RAST)—an eight-item questionnaire routinely used at Kenyan public clinics to determine HIV risk and PrEP eligibility (24). This tool includes questions on clients' HIV status and that of their partner(s), as well as their sexual history in the past 6 months, including history of sexually transmitted infections, needle sharing, sexual assault, and PEP usage. We then modified this tool for this pilot to ask an additional four questions about clients' exposure to HIV in the past 72 h and anticipated risk behaviors in the coming month. If a client answers yes to any of the screening questions, they will be potentially eligible for online HIV prophylaxis.

#### HIV testing

Clients identified as eligible for online HIV prophylaxis *via* the online self-screening tool will be directed to purchase a blood-based HIVST kit *via* MYDAWA to confirm their HIV-negative status prior to any prophylaxis initiation. Clients may choose between two different blood-based HIVST kits. The HIVST kits will be subsidized, and clients will be charged 349 Kenyan Shillings (KES) (~$3.50 US Dollars [USD]) for online delivery of an HIVST kit: 250 KES (~USD 2.50) for the kit and 99 KES (~USD 1.00) for the delivery fee. A MYDAWA pharmaceutical technologist will deliver this HIVST kit to clients at their desired location and provide pre-test counseling and instructions on the self-test use and interpretation of results at the point of delivery if requested.

To continue with the online PrEP/PEP delivery process, clients will need to upload an image of their self-test result and self-interpretation of this result *via* the MYDAWA platform (instructions on these steps will be delivered with the HIVST kit). Further instructions will be available on the MYDAWA platform. The user-friendly instructions and HIVST upload platform were developed by Audere (www.auderenow.org), a digital health nonprofit. Audere developed custom artificial intelligence algorithms which leverage computer vision and machine learning to ensure the uploaded image contains an HIV self-test result and is of high quality. For example, if the image is blurred or does not meet orientation and proximity expectations, the user interface will suggest that clients take a new image of the HIVST result to re-upload *via* the online platform.

#### Medical eligibility assessment

After HIV risk screening, clients can book a telemedicine consultation with a MYDAWA clinician using their unique user account ID *via* the online platform. This secured and deidentified ID links clients to their MYDAWA visits, allowing the researchers to track clients' PrEP follow-up visits over time. During the telemedicine visit, MYDAWA clinicians will review the HIV risk self-screening assessment completed by clients and the image of the client's uploaded HIVST result. Then, based on the client's exposure to HIV in the past 72 h, the clinician will determine if the client is best suited for PEP or PrEP. Clients who are eligible for PrEP/PEP but have not uploaded their HIVST results will be asked to upload their test results and book another telemedicine appointment once they have done so.

MYDAWA clinicians will have access to a decision support package to facilitate their interpretation of the clients' HIVST results and inform PrEP/PEP prescribing. The support package will include an image of the test captured by the client, the client's interpretation of the self-test, and three artifacts provided by Audere's algorithms: (1) an indication if a control or test line is present on the HIVST, (2) a cropped image of the HIVST result window, and (3) an enhanced view of the HIVST result window which attempts to highlight faint test lines. Clients whom MYDAWA clinicians determine self-tested HIV positive will be referred to treatment services at public or private healthcare facilities that meet their preferences.

Clients whom MYDAWA clinicians determine self-tested HIV negative will answer questions about medical conditions that might contraindicate PrEP safety (i.e., kidney or liver disease, diabetes, hypertension, or signs of acute HIV infection). Thus, MYDAWA clinicians will verify online HIV prophylaxis eligibility. Clients who report any medical conditions that might contraindicate PrEP safety will be referred to public or private clinics (client's choice) for in-person PrEP care by a clinical provider.

Clients who are confirmed HIV negative, at HIV risk, and medically eligible for PrEP or PEP by a MYDAWA clinical provider will be sent a link for an electronic consent form by the provider. This link will be sent to the client's phone or computer *via* short message service (SMS) or email. Once informed consent has been signed by the client, MYDAWA clinicians will prescribe PrEP or PEP and add the appropriate drug supply (30-days at PrEP initiation, 90-days at PrEP continuation, 28-days at PEP initiation) to the client's MYDAWA e-shopping cart.

#### HIV prophylaxis delivery

Clients determined eligible for online PrEP/PEP by the MYDAWA clinician may choose to order PrEP or PEP drugs *via* MYDAWA in the quantity associated with initiation or continuation visits. For this pilot study, the Kenya Ministry of Health is providing all PrEP and PEP drugs for free, and clients will only be charged a flat-rate 99 KES fee (~USD 1.00) for drug delivery services. Thus, this drug delivery fee will remain the same even as the volume of PrEP drugs may vary from initiation to refill visits. In addition, we will use pharmaceutical technologists to deliver PrEP and PEP drugs at the clients' preferred location and provide additional counseling on drug use and side effects at the point of delivery, as needed.

#### PrEP online refill

At the end of the telemedicine consultation, the clinician will inform clients that they will receive a PrEP refill reminder two days before their anticipated refill date *via* a secure email and encrypted text message. Clients must log in to the MYDAWA platform using their unique user account ID and repeat the HIVST process and medical safety assessment (via a telemedicine consultation) to complete the PrEP refill process.

#### PEP completion

All clients prescribed PEP will also be reminded to repeat HIVST at 1 month and 3 months post PEP prescription per Kenya Ministry of Health guidelines (25). At the one-month post-PEP prescription, the MYDAWA clinician will encourage those clients who are negative for HIV and at high risk for HIV acquisition to transition to PrEP if medically eligible.

#### PrEP or PEP support

Support options will include a call center and a MYDAWA chatbot feature to discuss PrEP or PEP concerns and potential side effects. Additionally, clients can request a telemedicine consultation with a clinical officer.

### Pilot data collection

The study team will obtain de-identified client-level data from MYDAWA *via* a secure data-sharing platform. This will include clients' age, sex, behaviors associated with HIV risk (i.e., responses to the modified 12-question RAST), HIV status (self-reported and confirmed *via* HIVST), and medical history (i.e., responses to questions asked by the MYDAWA clinician during the telemedical consultation). MYDAWA will also share de-identified data about all enrolled clients' purchases and deliveries related to sexual and reproductive health and online HIV prophylaxis delivery (e.g., HIVST, PrEP, and PEP dispensing). Additionally, de-identified data on clients' use of PrEP and PEP support tools (e.g., chatbot use and frequency and duration of calls to the MYDAWA call center) will be shared by MYDAWA with the study team.

### Pilot outcomes

#### Utilization and process outcomes

Our primary pilot outcomes will be PrEP and PEP initiation at 1 month among MYDAWA clients screened and determined at HIV risk and medically eligible for online HIV prophylaxis service delivery and PrEP continuation at 1 month among clients that initiated online PrEP (see Table 1 for details). Secondary outcomes will include PEP-to-PrEP transition and additional PrEP continuation outcomes (e.g., any continuation over the study period, ≥2 refills among those eligible). We will also measure PrEP stopping and restarting, defined as having a lapse in PrEP use for >14 days (26). Additionally, we will measure several process outcomes, including the percentage of clients who have HIVST delivered, uploaded the result of their self-test *via* the MYDAWA platform, correctly interpreted their self-test result, received reminders for PrEP refills, and used different MYDAWA support options (e.g., call center, telemedicine consultation, and chatbot) throughout their online HIV prophylaxis service delivery journey. Finally, throughout the pilot, we will carefully screen for and measure any social harms (e.g., gender-based violence) related to online PrEP and PEP delivery.

**Table 1:** The utilization outcomes, process outcomes, and client characteristics measured in the pilot study.

**Category**	**Outcome**	**Definition**	**Measurement**	**Timing**
Utilization outcomes	PrEP initiation^a^ (primary)	% of clients screened and determined at HIV risk and PrEP eligible *via* the online platform (MYDAWA) that initiated PrEP.	MYDAWA data; client surveys	Month 2
	PEP initiation (primary)	% of clients screened and determined at HIV risk and PEP eligible *via* the online platform (MYDAWA) that initiated PEP.	MYDAWA data; client surveys	Month 1.5
	PrEP continuation within 45 days of inititaion^b^ (primary)	% of clients who refilled PrEP *via* the online platform (MYDAWA) within 45 days of initiation among those who initiated PrEP.	MYDAWA data; client surveys	Month 2
	PEP to PrEP transition	% of clients screened and determined at HIV risk and PrEP eligible *via* the virtual platform (MYDAWA) that initiated PrEP after PEP completion.	MYDAWA data; client surveys	Month 1.5
	PrEP continuation with at least one refill^b^	% of clients who refilled PrEP *via* the online platform (MYDAWA) among those who initiated PrEP.	MYDAWA data; client surveys	Months 2 & 6
	PrEP continuation with at least 2 refills^b^	% of clients who refilled PrEP at least twice *via* the online platform (MYDAWA) among those who initiated PrEP.	MYDAWA data; client surveys	Months 2 & 6
	PrEP stopping and restarting	% of clients who refilled PrEP *via* the online platform (MYDAWA) with a gap of >14 days in pill coverage among those who initiated PrEP.	MYDAWA data; client surveys	Months 2 & 6
Process outcomes^c^	HIVST utilization	% of clients screened and determined at HIV risk and PrEP eligible *via* the online platform (MYDAWA) that ordered an HIVST.	MYDAWA data	N/A
	HIVST image upload	% of clients who uploaded the image of the self-test result on the online platform (MYDAWA) among those that ordered an HIVST.	MYDAWA data	N/A
	HIVST interpretation	% of clients who correctly identified the presence of test and control lines on their self-test result on the online platform (MYDAWA) among those that ordered an HIVST.	MYDAWA data	N/A
	MYDAWA clinical officer utilization	% of PrEP clients that consulted with the MYDAWA clinical officer (including and excluding during the prescribing process); summary of consultation topics discussed	MYDAWA data	N/A
	Selection of PrEP support tools	% of clients that used chatbot and call center to ask PEP/PrEP-related questions	MYDAWA data	N/A
Client characteristics^c^	Demographics	Client demographics including age, sex, income, occupation, and other demographics	MYDAWA data; client surveys	Month 0
	Behaviors associated with HIV risk	% of clients reporting different demographics (e.g., marriage, SES) and sexual behaviors (according to Kenya's RAST tool)	MYDAWA data; client surveys	Months 0, 2, & 6
	History of PrEP use	% of clients that are: (1) first time PrEP users, (2) past PrEP users, or (3) current PrEP users.	MYDAWA data; client surveys	Month 0
	Contraceptive use	% of clients reporting contraception use/type; frequency of EC	Client surveys	Month 0

^a^For all enrolled clients, we will call them 2 months following enrollment to measure self-reported outcomes.

^b^To remind clients to refill PrEP via the online platform, we will use SMS/email reminders.

^c^The pilot process outcomes and clinical characteristics include but are not limited to those listed in this table.

#### Client characteristics

We will measure the demographic characteristics (e.g., age. sex, marital status) and behaviors (e.g., sexual behaviors, health-seeking behaviors) of clients using online PrEP and PEP to understand if this novel delivery model expands the reach of HIV prophylaxis services beyond clients already being reached at public healthcare facilities.

### Pilot data analysis

We will use descriptive statistics to summarize online HIV prophylaxis delivery utilization outcomes (e.g., PrEP initiation and continuation), process outcomes (e.g., HIVST uptake), and the characteristics of clients who initiate and do not initiate these online services. We will summarize these outcomes among all enrolled participants and key sub-groups of interest, such as sex groups, age groups, and groups that did and did not achieve different process outcomes (e.g., received email and SMS reminders and utilized support tools). Additionally, we will use bivariate and multivariate regression models to identify client characteristics associated with PrEP initiation, PEP initiation, PEP-to-PrEP transition, and any PrEP continuation.

In a secondary analysis, we will also summarize the characteristics of the “early adopters” (those who engage in online PrEP services closer to launch, i.e., within the first 6 months) vs. the “late adopters” (those who engage in online PrEP services later in the pilot, i.e., after the first 6 months) to help MYDAWA establish more targeted marketing strategies for potential clients who fall later in the adoption continuum. All quantitative analysis will be completed in SAS, R, or STATA.

### Assessment of implementation outcomes

#### Acceptability and feasibility

We will assess the acceptability and feasibility (27) of an online HIV prophylaxis delivery model among clients and providers using behavioral questionnaires, in-depth interviews (IDIs), and routine implementation data (e.g., notes from meetings with the implementation team).

**Figure 2:**
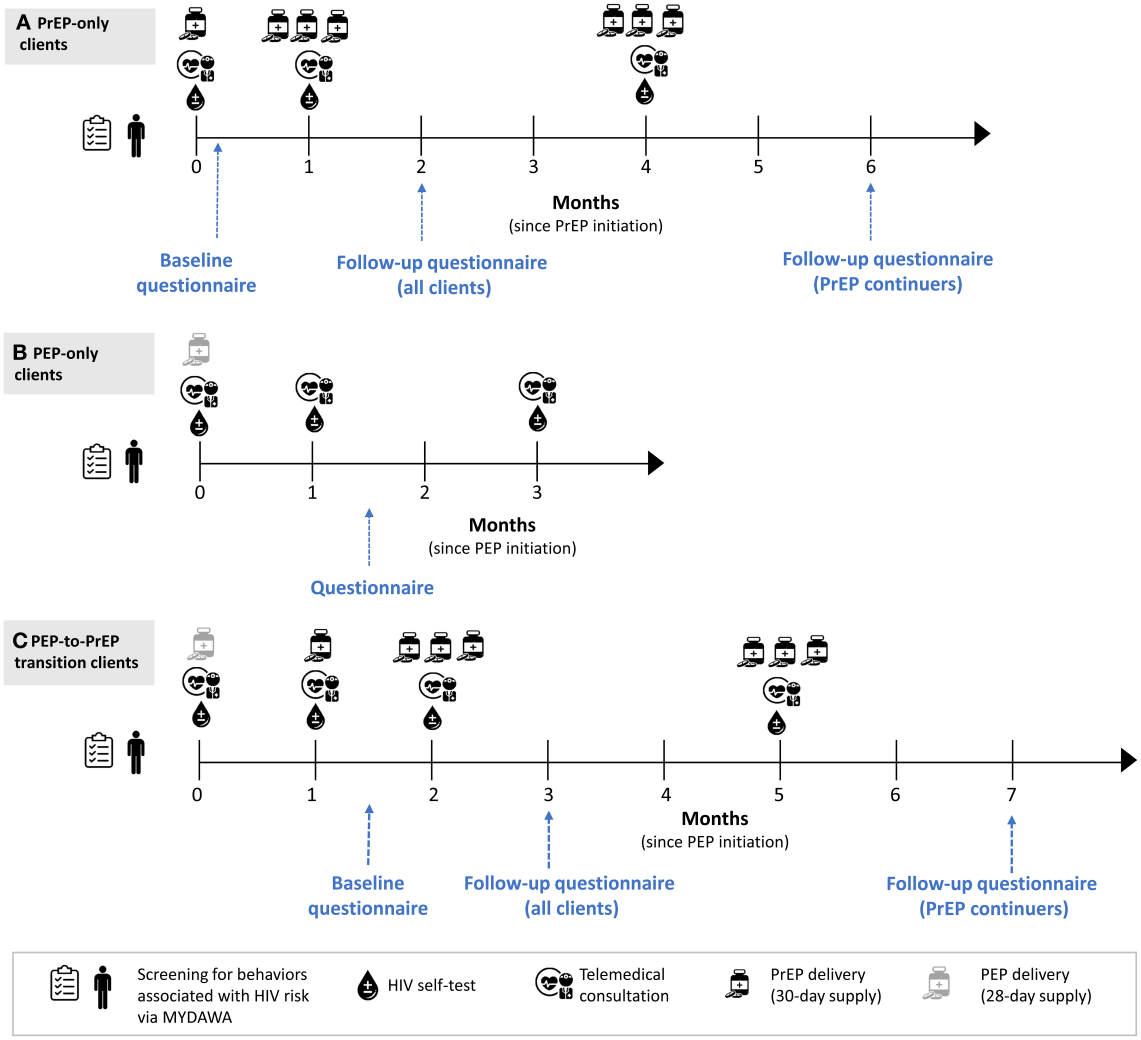
Overview of quantitative data collection at the client level. **(A)** PrEP only clients. **(B)** PEP-only clients. **(C)** Clients who transitioned from PEP to PrEP.

We will complete behavioral questionnaires with up to 500 clients enrolled in the study to understand their experiences with and perceptions of the intervention. This sample size is similar to other PrEP implementation studies that have measured like outcomes (28–30). A trained research assistant will call clients that have completed informed consent and invite them to complete a questionnaire that takes ~60 min. We will invite eligible clients to participate in these questionnaires right after enrollment and again at 2 and 6 months (PrEP clients) or 1.5 months (PEP clients) following enrollment (see Figure 2). Questionnaires will be conducted in-person or remotely, depending on the client's preference. Topics covered in the questionnaire include more detailed client demographics, assessment of clients' sexual behaviors, screening for the prevalence of depression, HIV prophylaxis use history, stigma associated with PrEP use, HIVST use history, adherence to HIV prophylaxis, potential side effects experienced, online HIV prophylaxis acceptability, willingness to pay for online HIV prophylaxis and social harms experienced during the study period (see Table 2 for details). Additionally, we will include quantitative assessments for online HIV prophylaxis acceptability.

**Table 2:** Behavioral data collection activities at enrollment (Baseline) and follow-up visits.

**Survey section**	**Description**	**Timing**	
		**Baseline**	**Follow up** ^a^
Demographics	Gender, age, education, monthly income, relationship status, marital satisfaction	X	
Sexual behaviors	Number of sexual partners, partners' HIV status, contraception use, if the client trying to conceive	X	X
Depression	Patient Health Questionnaire-2 (PHQ-2) tool	X	X
PrEP/PEP knowledge and use history	When and how the client learned of PrEP/PEP and online PrEP/PEP, prior PrEP/PEP use, source, reasons for stopping use, preference for where to obtain PrEP/PEP	X	
PrEP stigma	Client perceptions about PrEP stigma	X	
HIV testing history	prior HIV testing history, prior HIVST use history	X	
PrEP/PEP adherence	Number of PrEP/PEP pills missed in the past month, self-reported adherence quality, strategies used to remember taking pills	X	X
Potential drug side effects	Diarrhea, nausea, fatigue, aches	X	X
Online PrEP/PEP acceptability	Theoretical Framework of Acceptability (e.g., affective attitude, burden, perceived effectiveness)	X	X
Willingness to pay	HIVST, telemedicine, PrEP/PEP supply	X	X
Social harms	Gender-based violence and harms related to online PrEP/PEP service delivery	X	X

^a^Only PEP clients that transition to PrEP complete follow-up questionnaires.

Study staff will purposively identify (based on gender and age), contact, and invite clients and providers engaged in online HIV prophylaxis services to participate in IDIs that focus on the intervention-, client-, provider- and system-level factors influencing the implementation of the intervention (see Table 3 for details). We will conduct up to 100 IDIs, 80 IDIs with clients, and 20 IDIs with providers. All IDIs will be conducted in English or Swahili (the local language), either in-person or virtually, depending on the interviewee's preferences. The IDIs will last ~60 min and will be guided by the Theoretical Framework of Acceptability (31) to help us better define the multi-dimensional construct of acceptability, as well as the Structured Assessment of Feasibility (32) and the Health Belief Model (33), to help us identify potential barriers and facilitators of feasibility and behavior change. With permission, the IDIs will be audio-recorded. In addition, once a month, we will hold meetings with MYDAWA staff to discuss their primary responsibilities for this delivery model, challenges faced, and opportunities to improve PEP/PrEP delivery *via* this platform using structured guides. With permission, the discussion will be audio-recorded.

**Table 3:** Description of implementation outcome measures in the pilot study.

**Outcomes**	**Definition^a^**	**Measurement approach**	**Level of analysis**
		***Sample questions***	
Acceptability	The perception among stakeholders that online PrEP/PEP delivery is agreeable, palatable, or satisfactory.	Quantitative questions for clients based on the Theoretical Acceptability Framework (5-point Likert scale):	Client-, provider- and systems-level
		“*I like getting PrEP/PEP through MYDAWA” “I would like to continue getting PrEP/PEP through MYDAWA”*	
		Qualitative questions for clients:	
		What did you ^**^like^**^ about getting PrEP/PEP from MYDAWA? What did you ^**^dislike^**^ about getting PrEP/PEP from MYDAWA?	
		Qualitative questions for providers:	
		*What are your thoughts on how this program might be effective in providing HIV services for customers not reached by traditional methods?*	
Feasibility	The extent to which online PrEP/PEP delivery can be successfully implemented at scale in Kenya.	Qualitative questions for providers:	Provider- and systems-level
		*Do you think it is possible to implement online pharmacy services in Kenya to deliver PrEP/PrEP? Tell me more about that*.	
Costs	The incremental cost of implementing online PrEP/PEP delivery in Kenya.	*Micro-costing, staff interviews, and time and motion observation to estimate the financial and economic costs of implementing online PrEP/PEP delivery*.	Client-, provider- and systems-level
		Willingness to pay question for clients:	
		*Approximately how much would you be willing to pay for a remote/online clinical encounter to start PrEP/PEP services? (in Kenyan Shillings)*	

^a^Definitions derived from Proctor's framework of seven implementation outcomes (27).

We will use descriptive analysis to summarize the proportion of MYDAWA clients who reported that the online HIV prophylaxis delivery was acceptable, according to the assessments included in our questionnaires. Then we will use inductive and deductive qualitative analysis approaches and data from the IDIs to identify key themes related to the determinants of the intervention's acceptability and feasibility as well as any barriers and facilitators to implementing the online HIV prophylaxis delivery model. We will summarize key themes and relevant codes in a spreadsheet matrix guided by the socio-ecological framework (i.e., with client-, provider-, and system-levels) (34) and explore how these themes vary across these different levels. Additionally, to document any modifications that may occur to the online PrEP and PEP delivery model during implementation, we will use the Framework for Reporting Adaptations and Modifications to Evidence-based Implementation Strategies (FRAME-IS) (25). Finally, we will use Dedoose (https://www.dedoose.com/) to support the review and coding of all IDI transcripts and MYDAWA staff meeting minutes.

#### Costs

We will conduct micro-costing, staff interviews, and time-and-motion observation to estimate the financial and economic costs of implementing online PrEP and PEP delivery. Costs will be collected from the payer perspective. A trained research assistant will utilize standardized Excel cost menus to collect intervention costs: including start-up, software development, training, space, human resources, and supplies/equipment. Capital and start-up costs will be annualized, assuming a useful life of 5 years and a discount rate of 3%. The research assistant will interview staff and providers to assess the daily responsibilities of implementing online PrEP and PEP delivery. Time-and-motion observation of intervention activities from user/provider interactions (telemedicine visits with MYDAWA clinicians, delivery of HIVST by pharmaceutical technologists) will be used to measure staff time costs. Research time (e.g., administering informed consent) and other research costs will be removed from programmatic costs.

We will estimate the total annual cost and cost per client-initiated on PrEP using uptake data from the pilot and compare this to other models of PrEP delivery, including the standard-of-care at public clinics and the newly developed model of pharmacy-based PrEP delivery (22). Cost per client will be calculated as the total annual cost divided by the number of clients initiated on PrEP. We will estimate the costs of different operational components of online PrEP delivery, including demand generation approaches and HIV testing modalities. We will conduct scenarios and sensitivity analyses to assess the impact of various assumptions (e.g., client volume and scale, HIV testing strategies, demand generation approaches, and cost-sharing models among providers, payers, and clients).

## Discussion

This study will evaluate the initiation and continuation of PrEP and PEP, the feasibility and acceptability of an online PrEP and PEP delivery model, describe the characteristics of clients obtaining PrEP and PEP online, and evaluate the costs and implementation factors associated with online HIV prophylaxis delivery in Kenya. The evidence from this study will help us better understand who might be reached with an online model of PrEP and PEP service delivery, what their engagement in care might look like over time, and how much this new service delivery model might cost; information that can inform guidelines on online HIV prophylaxis delivery in Kenya and similar settings.

This study has several limitations. First, our study population is limited to individuals with computer or phone access who have sufficient technology and English language literacy to navigate an online pharmacy platform. Similarly, our study population is limited to individuals with the financial resources to pay for HIVST kits and PrEP or PEP medication delivery. Therefore, our findings may not be generalizable to individuals who do not have the technological literacy to use a platform like MYDAWA or do not have the financial resources to pay for the convenience of online service delivery. Third, due to the short follow-up period, we will not measure longer-term implementation outcomes (27). Despite these limitations, we anticipate that we will still be able to identify individuals who are not currently engaged with PrEP or PEP services, have unmet needs for these medications, and may use the online platform to access them.

Policy changes would need to occur for this model to be scalable and sustainable beyond this research study and frameworks for public-private partnerships would need to be established. For example, in Kenya and many other settings, HIVST is only recommended as a screening test, not to inform prescribing and dispensing of antiretrovirals (25, 35). In addition, while there has been progressive growth of telemedicine in Kenya, especially since the start of the COVID-19 pandemic, national guidelines for HIV services are premised on in-person care. Uploading a photo of an HIV test result to inform remote prescribing of antiretrovirals is new and some regulations around this process may need to be established. In this study, public-sector goods (e.g., PrEP and PEP drugs) were also provided for free to a private-sector company (e.g., MYDAWA) to help keep the price of online PrEP and PEP service delivery affordable to interested clients. For these public-private partnerships to be sustained, frameworks are needed to guide this collaboration and inform the amount private companies can charge for public goods. Additionally, record systems will need to be established to track the delivery of public commodities in private settings, among other things.

As access to telecommunications and new technologies continue to rise in sub-Saharan Africa (36), technology-based interventions, such as online HIV prophylaxis delivery, have great potential to expand the access of existing services to new populations and relieve overcrowded public healthcare facilities. This novel model of online PrEP and PEP delivery integrates telemedicine, at-home HIVST, and courier delivery to support at-home PrEP/PEP initiation and PrEP continuation, the first of its kind in Africa. This research will inform and potentially expand the list of HIV prophylaxis delivery models to help maximize the impact of these interventions and end the AIDS epidemic (37).

## Ethics statement

This research protocol has been reviewed and approved by the Scientific and Ethics Review Unit at the Kenya Medical Research Institute (Nairobi, Kenya) and the Institutional Review Board at the University of Washington (Seattle, USA). All research participants will provide electronic documents of written informed consent to participate in this study.

## Author contributions

KFO, KN, MLM, MS, and AS conceptualized this study. CK, PN, JCD, RCM, and MR were responsible for project administration. CK, PN, KFO, JCD, MLM, and AS completed writing. KFO, KN, and MLM provided supervision and acquired funding. All authors edited and revised the paper. All authors contributed to the article and approved the submitted version.
